# Development of a scale to measure selection, optimization, compensation (SOC) strategy in late middle-aged women: a methodological study

**DOI:** 10.4069/whn.2024.08.10

**Published:** 2024-09-30

**Authors:** Do-Young Lee, Gie Ok Noh

**Affiliations:** 1 Department of Nursing, Changshin University, Changwon, Korea; 2 Department of Nursing, Konyang University, Daejeon, Korea

**Keywords:** Instrumentation, Middle age, Strategy, Validation study, Women

## Abstract

**Purpose:**

Selection-optimization-compensation (SOC) models have been proposed and applied to various populations to examine successful aging from a multidimensional perspective. This study aimed to develop a scale to measure SOC strategy among late middle-aged women (aged 50 to 64 years) and to test its validity and reliability.

**Methods:**

Preliminary items were developed through a literature review and interviews. Overall, 32 preliminary items were confirmed via two rounds of expert content validity analysis and a pilot survey. Data were collected from 299 late middle-aged women and analyzed using IBM SPSS/PC+ version 27.0. Construct validity, criterion validity, and reliability tests were conducted.

**Results:**

The SOC strategy scale, reflecting the characteristics of late middle-aged women and developed through exploratory factor analysis, comprised 19 items across four factors: goal-oriented selection, compensation for loss, outcome optimization, and ability-based optimization. The scale explained 66.9% of the variance in total factors, with a Cronbach’s α of .95. Statistically significant correlations with the reference scale (r=.30, *p*<.001) were observed.

**Conclusion:**

The developed scale demonstrated high validity and reliability, thus representing a viable instrument for measuring SOC strategy among late middle-aged women. Using this scale to assess the use of SOC approaches in these women can improve our understanding of the aging process and help establish supportive programs for their aging journeys.

## Introduction

Advances in scientific and healthcare technologies have increased the average lifespan while extending the period of old age. To promote a smooth transition into this period, increasing attention has been devoted to successful aging after midlife [1]. A positive outlook on the aging process during midlife, which is a natural phase of the life cycle, is essential for successful aging and positive adaptation [2]. Assessing the aging process is particularly important for middle-aged women, who may experience a sudden onset of physical and emotional symptoms following menopause [3].

Successful aging involves not just physical health, but also psychological and social well-being, along with high life satisfaction [4]. The selection-optimization-compensation (SOC) model has been applied to facilitate favorable aging from a multidimensional perspective [5]. Designed for individuals who may feel isolated due to age, the SOC strategy involves choosing activities that suit their abilities (selection), working to perform these activities to the best of their ability (optimization), and using available resources to make up for limitations (compensation) [4]. Accordingly, this strategy is focused on positively adapting to the physical and environmental changes associated with aging and maximizing one’s available abilities, rather than attempting to overcome changes or dwelling on diminished capacities [6].

The SOC strategy scales used in South Korea (hereafter referred to as Korea) include a 48-item instrument developed by Baltes et al. [7], which was adapted and validated by Eom and Chung [8], along with a shortened 12-item version utilized by Song [9] and an extended 20-item version created by Sohn [10] that incorporated in-depth interviews with older adults. However, these tools employ dichotomous scales, presenting options as target and distractor items, which could lead to a polarized evaluation of respondent behavior. According to prior research, psychometric instruments assessing attitudes and behaviors should present individual situations and allow for selection based on specific circumstances [11]. Consequently, instruments must be developed using a Likert-scale format; this enables individuals to rate the alignment of each option with their typical behavior in a nuanced manner, without referencing specific situations.

Furthermore, since current scales for SOC strategies were developed for the elderly, items must be added or modified to cover the late middle-aged population (aged 50 to 64 years), who are faced with the onset of aging. Accordingly, a scale should be developed to appropriately measure SOC strategies to support a successful transition from middle to old age. Recent research on aging suggests that preparations for old age should be approached from a life-cycle perspective [12], and the outcomes and processes of successful aging must be measured during the period prior to old age. Middle age, a crucial period of preparation for successful aging, decisively influences the quality of life in later years [13]. Middle age is often broadly defined as an age of 40 to 64 years; however, for women, the hormonal changes experienced during this time may necessitate distinct approaches between early and late middle age [14]. Aging-related strategies likely differ between these stages.

The health crises, psychological changes, and coping behaviors of late middle-aged women during menopause represent key nursing concerns in the context of transitioning to old age [2]. However, suitable instruments are lacking for evaluating SOC strategies in light of these characteristics. As aging accelerates with the onset of menopause, an objective assessment of SOC approaches in late middle-aged women is crucial to establish interventions that support successful aging. Therefore, this study was performed to develop a scale to measure SOC strategies in late middle-aged women, drawing on the model proposed by Baltes et al. [7].

## Methods

**Ethics statement:** This study was approved by the Institutional Review Board of Konyang University (No. 2023-05-009-001). Informed consent was obtained from the participants.

### Research design

This methodological study was designed to develop a scale measuring SOC strategies in late middle-aged women. Furthermore, it aimed to evaluate the reliability and validity of the scale, adhering to the instrument development and validation processes proposed by DeVellis [15].

### Research procedure

**Figure 1.**
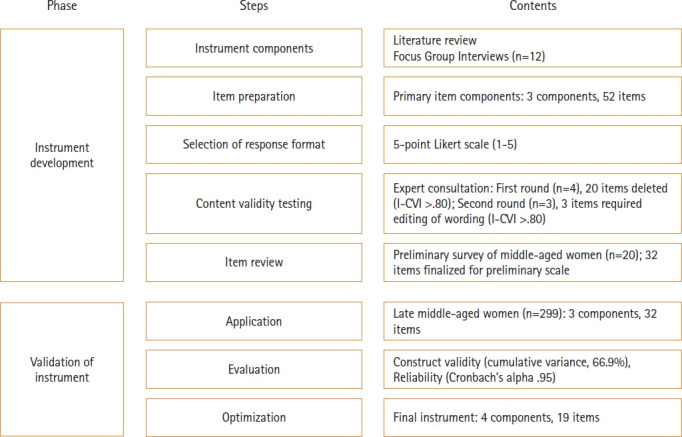
Scale development process. I-CVI: Item content validity index.

The study was conducted in two stages following the procedures proposed by DeVellis [15], as detailed in Figure 1.

#### Instrument development phase

##### 1) Instrument components

Before formulating the preliminary items, we established a conceptual framework that considered the attributes and components of the SOC model, as identified through a literature review and focus group interviews. This framework was designed to clarify concepts associated with the components of the SOC strategy proposed by Baltes and Baltes [4]. The model suggests that aging is an adaptive process involving three key components: selection, optimization, and compensation. Even in the face of age-related declines, individuals can experience successful aging by selecting activities suitable for their capabilities, optimizing their abilities, and compensating for deficits [6].

For the literature review, academic journals were searched using national and international databases such as PubMed, Google Scholar, Embase, CINAHL, KISS, KoreaMed, and DBpia. The search terms “SOC strategy” and “successful aging” were applied to publications from 1990 to 2022. In total, 498 articles were retrieved. After removing 124 duplicates and articles with topics irrelevant to the scope of the study, the remaining 374 articles underwent abstract and full-text screening. This process yielded 16 articles eligible for the final analysis.

To develop items for assessing SOC strategies in late middle-aged women, three focus group interviews were conducted with a total of 12 participants (four per group). Participants were recruited based on their age through snowball convenience sampling at the Women’s Education Center in Changwon, facilitating the collection of rich and meaningful data by encouraging interaction focused on the study topic. The semi-structured interview questions posed to the participants included: “What strategies do you believe are necessary for successful aging?” “How do you prefer to set goals for successful aging?” “What strategies can be used to minimize impairments and optimize abilities for successful aging?” and “What models are available to compensate for deficits in achieving successful aging?” Prior to the interviews, participants provided written consent, and the discussions were held in a private conference room, lasting approximately 2 hours. Two moderators conducted the interviews, and content analysis was performed on the transcripts. Drawing on insights from the literature review and the focus group interviews, preliminary items were formulated. These comprised 102 items, proportional to the numbers of themes and topics identified for each component of the SOC model.

##### 2) Item preparation

In this phase, duplicate items were removed, and the remaining items were categorized through a clustering process. This task was facilitated by a nursing professor and a professor with experience in instrument development. As a result, 52 items were organized into three distinct domains.

##### 3) Selection of a response format

Although a 4-point Likert scale is generally recommended to avoid central tendency bias, we adopted a 5-point Likert scale based on the suggestion that such an instrument is suitable for measuring attitudes and perceptions [15]. Our scale ranged from 1 (strongly disagree) to 5 (strongly agree), with a higher total score indicating a more favorable use of the SOC strategy.

##### 4) Content validity testing

The content validity of the initial items was assessed over two rounds by expert panels, following the recommendation of three to 10 experts for such evaluations [16]. The first-round panel consisted of two professors with expertise in instrument development and research on middle-aged women, as well as two professors with specializations in adult and women’s health nursing. The second panel comprised one professor experienced in instrument development and two professors specialized in women’s health nursing. Each item was evaluated using a structured 4-point Likert scale, and the questionnaire was designed to facilitate expert commentary and suggestions for revisions.

According to Lynn [16], an item content validity index (I-CVI) of 1.00 is necessary for appropriate content validity when evaluated by three to five experts, and a minimum of .78 is required when assessed by six to 10 experts. In the first round, 30 items with an I-CVI below 1.00 were discarded. Subsequent feedback prompted revisions and reorganization, yielding a revised set of 32 items. The second round revealed no items with an I-CVI below .78; however, expert feedback indicated that two items were redundant and one needed rephrasing, leading to the revision of three items.

These efforts yielded 32 preliminary items. A pilot survey was then conducted with 10 late middle-aged women recruited through the Women’s Education Center. The survey assessed the clarity of sentence construction, the placement of items, and any potential ambiguities. Participants took an average of 10 minutes to complete the questionnaire. While no difficulties in responding were observed, feedback included a suggestion to avoid the repetitive use of “I am” at the beginning of each item. As a result, this phrase was positioned at the top of the questionnaire to apply to all items. This change was enabled by the syntax of the Korean language, which typically omits personal pronouns when the context is clear.

##### 5) Item review

The SOC strategy scale, now tailored for late middle-aged women, was finalized with 32 items distributed across three domains: selection (10 items), optimization (10 items), and compensation (12 items). This scale formed the survey for the main study.

#### Instrument validation phase

Item, exploratory factor, validity, and reliability analyses were conducted on the 32 selected items to validate their construct validity, criterion validity, and reliability.

##### 1) Participants

This study targeted late middle-aged women in Korea. Participants were recruited using convenience and snowball sampling methods, with recruitment notices posted on websites popular among this population. The inclusion criteria required participants to be late middle-aged women between 50 and 64 years old, without self-reported cognitive impairment, who possessed communication and reading comprehension skills, understood the purpose of the study, and voluntarily agreed to participate.

The validity of the preliminary instrument developed in this study was statistically tested using model fit analysis via structural equation modeling. Khine [17] states that a minimum of 150 participants is necessary to employ the maximum likelihood method in structural equation modeling. Additionally, DeVellis [15] recommends that the sample size for exploratory factor analysis be 5 to 10 times the number of items developed. Accounting for an anticipated 10% dropout rate [18], the survey was distributed to 300 individuals. Following the exclusion of one incomplete response, 299 responses were ultimately analyzed.

##### 2) Data collection and ethical considerations

Data were collected online from November 2023 to January 2024 using Google Forms. Participants were directed to the survey via a URL provided in the recruitment notice. The survey targeted late middle-aged women who met the inclusion criteria and provided voluntary informed consent. They were fully briefed on the study’s purpose, rationale, procedures, and methods; the estimated time required to complete the questionnaire; the confidentiality protocols for personal information; and their right to withdraw from the study at any time. The collected data were assigned individual identification numbers and protected with passwords to prevent the disclosure of personal information. As an incentive for their participation, respondents were given a mobile voucher valued at approximately 2 US dollars.

##### 3) Measurements

For criterion validation of the 32-item instrument developed in this preliminary study, a modified version of the SOC strategy instrument proposed by Baltes et al. [7] was used. The general characteristics of the late middle-aged women who participated in the study were assessed, including age, occupation, religion, education level, marital status, economic status, chronic diseases, self-perceived health status, life satisfaction, and menopause status. The criterion instrument, originally designed to measure the extent of SOC utilization, had been modified and shortened by Song [9]. This version was used after permission was obtained from the original author. It consists of 12 items, each framed as a choice between a target and a distractor. Respondents select the option that better reflects their attitudes or behaviors. Each target item selected earns one point, while distractor items receive zero points, resulting in a total score that can range from 0 to 12. A higher score indicates a greater use of SOC strategies. In the study proposing the tool [7], the overall item reliability was not reported, and the subscale reliability Cronbach’s α values ranged from .61 to .65. In Song’s report [9], the overall item reliability, as measured using Cronbach’s α, was reported to be .70.

##### 4) Data analysis

The collected data were analyzed using IBM SPSS/PC+ version 27.0 (IBM Corp., Armonk, NY, USA). The general characteristics of the participants were assessed using descriptive statistics, including frequency, percentage, mean, and standard deviation. For item analysis, the skewness and kurtosis values were checked; initially, items with absolute skewness values greater than 3 and kurtosis values greater than 7 were eliminated. Subsequently, items that correlated with a total score of less than .3 were also removed, as suggested by Boateng et al. [19].

The Kaiser-Meyer-Olkin (KMO) test and the Bartlett test of sphericity were conducted to assess the suitability of the data for exploratory factor analysis. The KMO value exceeded .80 and the Bartlett test indicated significance (*p*<.05), both supporting the data’s appropriateness for analysis. Subsequently, exploratory factor analysis was performed using maximum likelihood extraction and varimax rotation. Only factors with eigenvalues greater than 1.0 were retained as subfactors of the instrument. Additionally, items with a factor loading of .50 or higher were included, while items demonstrating cross-factor loadings below .40 were excluded.

The criterion validity of the instrument was assessed by analyzing its correlation with a reference instrument. Reliability was verified by calculating Cronbach’s α for the overall instrument and assessing the impact on this α value when individual items were removed.

## Results

### General characteristics of the participants

The study included 299 participants with an average age of 53.44±3.56 years, ranging from 50 to 64 years. Most participants were employed (n=231, 77.3%), married (n=249, 83.3%), and religiously affiliated (n=266, 89.0%). The most frequent level of educational attainment was a bachelor’s degree (n=168, 56.2%), and the predominant economic status was the middle level (n=202, 67.5%). Overall, 183 participants (61.2%) reported having no chronic diseases, and 163 individuals (54.5%) rated their health status as average. Just over half of the participants (53.5%, n=160) expressed satisfaction with their lives, and 197 participants (65.9%) were postmenopausal (Table 1).

### Item analysis

The normality of the data collected for item analysis was confirmed by examining skewness and kurtosis values, which ranged from −1.06 to .01 and −.59 to 1.52, respectively. Since none of the items exhibited absolute values exceeding 3 for skewness or 7 for kurtosis, no items were excluded based on these criteria. Further analysis revealed that six items (items 3, 17, 25, 27, 28, 31) had correlation coefficients below .30, prompting their removal. After re-evaluating the correlations, the values obtained ranged from .31 to .82. Ultimately, 26 items were retained for exploratory factor analysis to assess construct validity.

### Construct validity testing

Exploratory factor analysis was employed to assess the construct validity of the instrument developed to measure SOC coping strategies among late middle-aged women. The data’s suitability for factor analysis was previously established, evidenced by a KMO value of .90 and a Bartlett test of sphericity indicating significance (*χ*^2^=6,341.23, *p*<.001). Exploratory factor analysis was performed using maximum likelihood extraction and varimax rotation. Communalities for the individual items ranged from .43 to .82. Four items with communalities below .50 were excluded: items 5 (.47), 14 (.38), 26 (.43), and 29 (.45).

After eliminating these four items, a second-factor analysis was conducted. Items 4 and 32, which had respective communalities of .31 and .49, were excluded prior to a third-factor analysis. In this latter analysis, all remaining items had communalities greater than .50, resulting in a set of 20 items.

The third-factor analysis yielded four factors with eigenvalues exceeding 1.0, accounting for a cumulative variance of 66.4%. Item 21, however, exhibited a factor loading below .50 and was thus excluded from this analysis. A fourth-factor analysis was subsequently performed with the remaining 19 items, identifying four factors with eigenvalues of 1.0 or greater and explaining a cumulative variance of 66.9%. In this final factor analysis, factor 1 accounted for 20.1% of the variance, factor 2 for 18.7%, factor 3 for 18.0%, and factor 4 for 10.2% (Table 2).

The exploratory factor analysis, conducted in three stages, resulted in the removal of seven items with communalities below .50. These items were: “I take the lead when making important life decisions,” “When I want to execute a plan, I consider the approaches of successful people,” “I obtain information on managing health changes from healthcare professionals,” “When things do not go well, I seek help or advice from others,” “If something no longer works like before, I try different methods until I achieve comparable results,” “If life is not going as well as it used to, I watch useful broadcasts or read books,” and “When things are unresolved, I continue trying different methods.”

### Criterion validity testing

A significant correlation with the reference instrument was confirmed [9], establishing the criterion validity of the SOC strategy scale (r=.30, *p*<.001).

#### Reliability testing

To evaluate the reliability of the final 19-item scale developed in this study, we assessed the reliability of the subfactors and the entire item set, as well as Cronbach’s α value, when items were removed. The overall reliability of the items was .95, while the reliability scores for the individual factors were .90 for factor 1, .88 for factor 2, .90 for factor 3, and .90 for factor 4. These results confirmed the scale’s suitability for use. Furthermore, the removal of items did not significantly affect the reliability scores, indicating that no additional items required exclusion (Table 3).

#### Finalization of the SOC strategy scale

After testing for validity and reliability, the SOC strategy scale developed for late middle-aged women consisted of 19 items. The items are grouped under four factors: factor 1, goal-oriented selection; factor 2, loss compensation; factor 3, outcome optimization; and factor 4, ability-based optimization (Table 3). Each item is rated on a 5-point Likert scale, with total scores ranging from 19 to 95. Higher scores signify greater application of SOC approaches (Supplementary Materials 1 [English version] and 2 [Korean version]).

## Discussion

We developed and tested a scale measuring the application of the SOC strategy, specifically for late middle-aged women. This work was based on the SOC model proposed by Baltes and Baltes [4]. The resulting instrument comprises 19 items divided into four factors: goal-oriented selection (four items), loss compensation (seven items), outcome optimization (six items), and ability-based optimization (two items).

Of the seven items removed due to communalities below .5 in the exploratory factor analysis, many share characteristics with SOC strategies commonly employed by older adults. These include seeking advice or assistance from others and turning to rest and physical care when confronted with limitations. Notably, middle age is a life stage marked by substantial roles and responsibilities, making individuals particularly susceptible to adverse life events and disabilities [20]. Additionally, middle-aged people are charged with contributing to societal productivity and guiding the next generation, a concept known as generativity strivings [21]. Consequently, productivity may facilitate a smoother transition for middle-aged individuals into a fulfilling old age. This pursuit can be viewed not as an event to be dreaded but rather as a proactive strategy to be embraced.

Factor 1, goal-oriented selection, includes four items and accounts for 20.1% of the observed variance. This factor relates to the methods used to pursue goals. In this context, “selection” is operationally defined as narrowing the scope of activities to areas in which high performance can be maintained, while disregarding other areas, as one grows older and experiences decline [6]. In the elderly, selection typically involves emphasizing or identifying goals from available options in response to limited personal resources [10]. However, unlike the elderly, who restrict their range of activities or exclude certain possibilities, late middle-aged women appear to opt to exhibit productivity through role fulfillment, while considering both present and future circumstances and setting priorities accordingly.

Factor 2, loss compensation, includes seven items and explains 18.7% of the variance. The items relate to approaches for addressing problems, such as difficulties in maintaining routine tasks. Compensation strategies include actions taken to mitigate the consequences of the biological and social impairments associated with aging [6]. Research indicates that late middle-aged women also adopt compensatory actions in response to changes and losses [22], and positive expectations about aging may alleviate aging anxiety among these women by addressing psychological elements. Factor 2 is characterized by various compensatory behaviors that individuals adopt to manage age-related changes. In Sohn’s research [10], which developed the short form of the SOC strategy scale for the elderly, compensation strategies involved seeking help from others or relying on faith. However, the present study reveals that late middle-aged women are distinct in their proactive approach to achieving goals through various challenging behavior strategies, such as focusing on work, establishing an optimal environment, collaborating with others, and unflinchingly engaging in new attempts. Notably, the items identified in this study include social relationship elements, such as “When things do not go as before, I collaborate with people who share the same goals.” This departs both from previous research that used structural equations to analyze SOC strategy relationship factors, in which social relationship variables were not considered [23], and from SOC strategy scales for the elderly that lacked social relationship items [7,9,10]. Social activities have been identified as a key component of successful aging in elderly individuals, positively impacting adaptability and resilience during crises through interactions with others [24]. For late middle-aged women facing the onset of aging, maintaining social activities and employing SOC strategies that involve social relationships are of even greater importance. This finding aligns with previous research [25] indicating that sustaining social relationships based on one’s social network can support life satisfaction, improve well-being, and increase productivity during middle age.

Factor 3, outcome optimization, comprises six items and accounts for 18.0% of the variance. These items primarily concern the adjustment of situations or psychological attitudes, as well as physical responses, to achieve the best results possible. Demonstrating a positive attitude and effectively managing stress are considered essential during middle age. This life stage is characterized by tasks associated with developing self-confidence, building personal skills, and fostering competence and positivity through broad social experiences [20]. The items pertaining to physical responses for outcome optimization suggest that adapting to physical changes is a key aspect for late middle-aged women. This finding corroborates research suggesting that cognitive function and functional health status are directly linked to successful aging in the elderly [23]. Middle age is often portrayed as a time of crisis due to the onset of physical decline and a reduction in abilities, which are not encountered in earlier developmental stages [13].

Factor 4, ability-based optimization, includes two items and explains 10.2% of the variance. This factor pertains to the strategic maximization of capabilities to fully realize one’s potential and maintain personal efficiency. The items include statements such as “I would like to further develop myself by learning the latest technologies along with the skills I already possess” and “I establish strategies to maximize my abilities.” These reflect the notion of ability-based optimization among late middle-aged women, as informed by their experiences.

This study has several limitations. First, the validity of the developed scale was assessed using exploratory factor analysis alone, without the support of confirmatory factor analysis. Second, the generalizability of the study results is limited, as participants were recruited through an online link posted on the websites of regional institutions. Therefore, follow-up studies are necessary to confirm the validity of the developed scale. Finally, this study focused on creating an SOC strategy scale tailored to late middle-aged women. Future research should aim to broaden the scope by developing measurement tools for various life stages and for both men and women.

The SOC strategy scale developed in this study facilitates an objective assessment of the aging process in late middle-aged women, who are experiencing menopausal symptoms and approaching old age. Furthermore, since SOC strategy scales for successful aging were previously available only for the elderly, this instrument can provide fundamental data supporting interventions to promote the successful aging of late middle-aged women.

## Figures and Tables

**Table 1.** General characteristics of participants (N=299)

Characteristic	Categories	n (%) or mean±SD
Age (year)		53.44±3.56
Employed	Yes	231 (77.3)
	No	68 (22.7)
Religion	Yes	266 (89.0)
	No	33 (11.0)
Education	≤High school diploma	37 (12.4)
	Bachelor’s degree	168 (56.2)
	≥Master’s degree	94 (31.4)
Married	Yes	249 (83.3)
	No	50 (16.7)
Economic status	High	89 (29.8)
	Middle	202 (67.5)
	Low	8 (2.7)
Chronic disease	Yes	116 (38.8)
	No	183 (61.2)
Perceived health status	Healthy	108 (36.1)
	Average	163 (54.5)
	Unhealthy	28 (9.4)
Life satisfaction	Satisfied	160 (53.5)
	Neutral	139 (46.5)
Menopause	Yes	197 (65.9)
	No	102 (34.1)

**Table 2.** Results of construct validity testing (N=299)

Item No.	Item	Com¬muna¬lities	Factor loadings			
			Factor 1	Factor 2	Factor 3	Factor 4
6	When I set goals, I consider future situations thoroughly.	.66	.68*	.20	.30	.26
7	When I am unable to perform tasks like I could before, I consider what is currently most important.	.75	.79*	.19	.28	.13
8	When things go awry, I start again while considering the most important goal.	.68	.72*	.34	.04	.20
10	When more effort is needed, I contemplate what I truly desire.	.78	.76*	.39	.21	.04
1	When I strive to achieve goals, I tend to focus on the most important ones.	.56	.27	.67*	.08	.14
2	I set clear goals and work toward achieving them.	.52	.25	.63*	.03	.23
8	When maintaining usual tasks becomes difficult, I focus my efforts on what is still feasible.	.57	.39	.57*	.29	-.05
11	I create an optimal environment for achieving predetermined goals.	.60	.39	.53*	.34	.24
12	When things don’t progress as before, I collaborate with individuals sharing the same goals.	.51	.25	.59*	.26	.21
13	I consider the most effective method for implementing plans.	.66	.47	.57*	.32	.12
18	I do not hesitate to try new approaches to achieve my goals.	.70	.01	.70*	.34	.31
15	I leverage my strengths when executing plans.	.79	.50	.20	.56*	.43
16	I consider the most suitable timing for executing plans.	.76	.48	.22	.66*	.20
22	Although it may be challenging to do things as before, I maintain a positive outlook in all situations.	.69	.12	.35	.71*	.19
23	I resort to my own methods to manage stress.	.69	.40	.19	.69*	.16
24	I monitor my body to detect physical changes or warning signs.	.60	.39	.28	.58*	.20
30	I adopt a regular lifestyle tailored to my changed health status without overexertion.	.50	.04	.06	.70*	.10
19	I desire to learn and develop further by acquiring the latest technologies alongside my existing abilities.	.74	.22	.43	.25	.66*
20	I establish strategies to leverage my abilities to the maximum.	.98	.23	.29	.33	.87*
Eigenvalue			3.81	3.55	3.42	1.93
Explained variance (%)			20.1	18.7	18.0	10.2
Cumulative variance (%)			20.1	38.8	56.7	66.9

* *p*<.05.

**Table 3.** Instrument reliability analysis (N=299)

Factor (number of items)	Item No.	Cronbach’s α for deleted item	Cronbach’s α
Factor 1: Goal-oriented selection (4)	6	.95	.90
	7	.95	
	8	.94	
	10	.94	
Factor 2: Loss compensation (7)	1	.94	.88
	2	.94	
	9	.94	
	11	.94	
	12	.94	
	13	.94	
	18	.94	
Factor 3: Outcome optimization (6)	15	.94	.90
	16	.94	
	22	.94	
	23	.94	
	24	.94	
	30	.94	
Factor 4: Ability-based optimization (2)	19	.94	.90
	20	.94	
Total	19 items		.95

## Supplementary materials

Further details on supplementary materials are presented online (available at https://doi.org/10.4069/whn.2024.08.10).

Supplementary Material 1. Selection-optimization-compensation (SOC) strategy scale for late middle-aged women (English version)

Supplementary Material 2. Selection-optimization-compensation (SOC) strategy scale for late middle-aged women (Korean version)
