# Supplementary material for: Development of a scale to measure selection, optimization, compensation (SOC) strategy in late middle-aged women: a methodological study
Source: Womens Health Nurs. 2024 Sep 30;30(3):216–25. doi: 10.4069/whn.2024.08.10 (PMC11467244; doi:10.4069/whn.2024.08.10)
Supplement: Supplementary Material 1. — Selection-optimization-compensation (SOC) strategy scale for late middle-aged women (English version) [file whn-2024-08-10-Supplementary-Material-1.pdf]

Supplementary Material 1. Selection-optimization-compensation (SOC) strategy scale for late middle-aged women (English version)

Attributes		Preliminary items	1	2	3	4	5
Goal-oriented selection	1	When I set goals, I consider future situations thoroughly.					
	2	When I am unable to perform tasks like I could before, I consider what is currently most important.					
	3	If things go awry, I start again while considering the most important goal.					
	4	When more effort is needed, I contemplate what I truly desire.					
Loss compensation	1	When I strive to achieve goals, I tend to focus on the most important ones.					
	2	I set clear goals and work toward achieving them.					
	3	When maintaining usual tasks becomes difficult, I focus my efforts on what is still feasible.					
	4	I create an optimal environment for achieving predetermined goals.					
	5	When things do not go as before, I collaborate with people who share the same goals.					
	6	I consider the most effective method for implementing plans.					
	7	I do not hesitate to try new approaches to achieve my goals.					
Outcome optimization	1	I leverage my strengths when executing plans.					
	2	I consider the most suitable timing for executing plans.					
	3	Although it may be challenging to perform tasks as before, I maintain a positive outlook in all situations.					
	4	I resort to my own methods to manage stress.					
	5	I monitor my body to detect physical changes or warning signs.					
	6	I adopt a regular lifestyle tailored to my changed health status without overexertion.					
Ability-based optimization	1	I would like to further develop myself by learning the latest technologies along with the skills I already possess.					
	2	I establish strategies to maximize my abilities.					

1: Strongly disagree, 2: disagree, 3: neutral, 4: agree, 5: strongly agree.

https://doi.org/10.4069/whn.2024.08.10
