# Supplementary material for: Development of a scale to measure selection, optimization, compensation (SOC) strategy in late middle-aged women: a methodological study
Source: Womens Health Nurs. 2024 Sep 30;30(3):216–25. doi: 10.4069/whn.2024.08.10 (PMC11467244; doi:10.4069/whn.2024.08.10)
Supplement: Supplementary Material 2. — Selection-optimization-compensation (SOC) strategy scale for late middle-aged women (Korean version) [file whn-2024-08-10-Supplementary-Material-2.pdf]

Supplementary Material 2. Selection-optimization-compensation (SOC) strategy scale for late middle-aged women (Korean version)

Attributes		Preliminary items	1	2	3	4	5
목표를 위한 선택	1	목표를 선택할 때, 앞으로의 상황을 충분히 고려한다.					
	2	전에 해오던 일을 할 수 없게 되었을 때, 현재 가장 중요한 것이 무엇인지를 고려한다.					
	3	일이 잘 풀리지 않으면 가장 중요한 목표부터 다시 추진해 본다.					
	4	더 많은 노력이 필요한 일이 발생했을 때, 내가 진실로 원하는 것이 무엇인지를 고려한다.					
상실에 대한 보상	1	원하는 것을 이루려고 할 때, 중요한 일에 집중하는 편이다.					
	2	목표를 명확히 세우고, 그 목표를 성취해 나간다.					
	3	평소 하던 일을 유지하기 어려워졌을 때, 아직 가능한 일에 노력을 기울인다.					
	4	정해진 목표를 달성하기 위해 내가 잘 할 수 있는 최적의 환경을 조성한다.					
	5	전처럼 일이 진행되지 않을 때, 같은 목표를 갖는 사람들과 협력한다.					
	6	계획을 실현할 수 있는 가장 정확한 방법이 무엇인지 고려한다.					
	7	목표 달성을 위해 새로운 시도를 실행하는 것을 두려워하지 않는다.					
결과의 최적화	1	계획을 실행할 때, 나의 강점을 충분히 활용한다.					
	2	계획을 실현하기에 가장 적합한 시기를 고려한다.					
	3	전처럼 하는 것이 어렵지만 모든 상황을 긍정적으로 바라본다.					
	4	스트레스를 조절하기 위해 나만의 방법을 사용한다.					
	5	신체적인 변화나 위험신호를 알기 위해 나의 몸을 관찰한다.					
	6	변화된 건강 상태에 맞추어 규칙적인 생활을 하며 무리하지 않는다.					
능력을 기반한 최적화	1	내가 가진 능력과 함께 최신 기술을 배워 좀 더 발전시키고자 한다.					
	2	내가 가진 능력을 최대한 발휘하기 위한 전략을 세운다.					

1: 전혀 그렇지 않다, 2: 그렇지 않다, 3: 보통이다, 4: 대체로 그렇다, 5: 매우 그렇다.

https://doi.org/10.4069/whn.2024.08.10
